# Biological Functionalities of Transglutaminase 2 and the Possibility of Its Compensation by Other Members of the Transglutaminase Family

**DOI:** 10.1155/2014/714561

**Published:** 2014-03-23

**Authors:** Benedict Onyekachi Odii, Peter Coussons

**Affiliations:** Biomedical Research Group, Department of Life Sciences, Faculty of Science & Technology, Anglia Ruskin University, East Road, Cambridge, CB1 1PT, UK

## Abstract

Transglutaminase 2 (TG2) is the most widely distributed and most abundantly expressed member of the transglutaminase family of enzymes, a group of intracellular and extracellular proteins that catalyze the Ca^2+^-dependent posttranslational modification of proteins. It is a unique member of the transglutaminase family owing to its specialized biochemical, structural and functional elements, ubiquitous tissue distribution and subcellular localization, and substrate specificity. The broad substrate specificity of TG2 and its flexible interaction with numerous other gene products may account for its multiple biological functions. In addition to the classic Ca^2+^-dependent transamidation of proteins, which is a hallmark of transglutaminase enzymes, additional Ca^2+^-independent enzymatic and nonenzymatic activities of TG2 have been identified. Many such activities have been directly or indirectly implicated in diverse cellular physiological events, including cell growth and differentiation, cell adhesion and morphology, extracellular matrix stabilization, wound healing, cellular development, receptor-mediated endocytosis, apoptosis, and disease pathology. Given the wide range of activities of the transglutaminase gene family it has been suggested that, in the absence of active versions of TG2, its function could be compensated for by other members of the transglutaminase family. It is in the light of this assertion that we review, herein, TG2 activities and the possibilities and premises for compensation for its absence.

## 1. Introduction

The human transglutaminase 2 (TGM2) gene localizes to chromosome 20q11-12 and its exons span approximately 37 kb [1]. The protein, transglutaminase 2 (TG2, EC 2.3.2.13), is made up of 687 amino acids, with molecular mass of 77.3 kDa [2, 3]. Transglutaminase 2 (TG2) is also known as tissue transglutaminase (tTG), cytosolic, type II, or liver transglutaminase. It is the most abundant and most studied of the nine members of the transglutaminase enzyme family, including TG1, TG3, and TG5 isoforms, which are predominantly expressed in epithelial tissue; TG4, which is expressed in the prostate gland; factor XIII (FXIII), which is expressed in the blood; TG6 which is expressed in the testis, lungs, and brain [4, 5]; TG7, which is ubiquitously expressed but most prominently distributed in the testis and lungs [4].

A further member of the TG2 family is band 4.2, which is an enzymatically inactive protein component of the erythrocyte membrane that shares homology with many transglutaminases but lost the characteristic transglutaminase activity as a result of an amino acid substitution (Cys-Ala) at the active site [4, 6]. It is expressed in the surface of erythrocyte membranes, bone marrow, foetal liver, and spleen and serves as a key component of erythrocyte skeletal network, where it maintains erythrocyte shape and mechanical properties [4, 6] (Table 1).

Furthermore, shorter forms of TG2 with different properties have been reported to be produced through the alternative splicing of transglutaminase 2 encoding gene (TGM2) [3, 7–9]. A total of four spliced forms of TG2 have been reported, including TG2-S, TG2-H2, TG2_*v*1_, and TG2_*v*2_, but their roles* in vivo* are yet to be defined (as reviewed by [10]). The major difference between TG2 and its spliced isoforms is that all the spliced isoforms lose their C-terminus to different extents; therefore, they cannot undertake the characteristic functions of TG2 like GTP/ATP binding, PLC binding, migration and adhesion functions, and so forth [11–13]. Though, some of these isoforms of TG2 are yet to be properly characterised, TG2 short isoform (TG2-S), which lacks the C-terminal 138 amino acid residues of full length TG2, has been characterised and reported to be upregulated in brain tissue of Alzheimer's patients [14–16].

Structurally, TG2 is similar to those of other members of its family, except that it bears some specific features which are not characteristic of other type of transglutaminases. Essentially, TG2 is composed of four distinct globular domains: A NH_2_-terminal *β*-sandwich which contains fibronectin and integrin binding sites, a catalytic core which contains the catalytic triads (Cys277, His335, and Asp358) for acyl-transfer reaction and a conserved tryptophan essential for this catalytic reaction [17, 18], and two COOH-terminal *β*-barrel domains with the second barrel domain containing a phospholipase C binding sequence [19, 20].

Unlike other transglutaminases, TG2 possesses a unique guanidine nucleotide-binding site, located in the cleft between the catalytic core and the first *β*-barrel [20]; this sequence is coded by exon 10 of the TG2 gene, which is characterised by very poor sequence homology with the same exons in other transglutaminases. Some GDP/GTP-interacting residues and those necessary for GTP hydrolysis are situated in other domains [21]. In the GDP-bound form of TG2, access to the transamidation active site is blocked by two loops, and the active site cysteine is attached to a tyrosine residue by hydrogen bonding. In the latent conformation of TG2, there is a significant interdomain interaction between the catalytic domain 2 and domains 3 and 4, which reduces the accessibility of the active centre [20].

Besides the primary transglutaminase enzymes' activity of catalyzing the calcium-dependent posttranslational modification of proteins, TG2 can also bind and hydrolyze GTP [22], exhibit protein disulphide isomerase activity [23], and function as a protein kinase independently of calcium [24]. Furthermore, TG2 has calcium-independent nonenzymatic activities, especially extracellularly, where it interacts with a number of cell surface proteins [25], taking part in cell adhesion processes and stabilization of the extracellular matrix. This catalogue of special activities and multiple functionalities is underlined by TG2's structural uniqueness and complexity typified by its complex four-domain structure, constituted of an N-terminal *β*-sandwich, a catalytic core, and two C-terminal *β*-barrel domains [18]. Regardless of the wide range of biological functionalities associated with TG2 activities, amidst its unique cellular biochemistry, its exact physiological function is still debated. Hence, the view that in the event of its absence, the physiological function of TG2 could be compensated for by another member(s) of the transglutaminase family. It is against this background, that we considered the implications of TG2 expression and TG2-specific activities in biological processes. This is done with a view to rationalizing its involvement in many cellular physiological events and dissecting the premise that its functional compensation is feasible or unlikely.

## 2. Transglutaminase 2-Specific Enzymatic Activities

Catalysis of Ca^2+^-dependent posttranslational modification of proteins is the major hallmark of transglutaminase enzymes. The mechanism of this reaction is generally the same for TG2 and other members of TG-enzyme family and involves a two-step process, as recently reviewed in Gundemir et al. [26]. The first step is the formation of a thioester bond with the enzyme's active cysteine site via the transamidation of the *γ*-carboxamide group of a peptide bond glutamyl substrate, which is accompanied by the release of ammonia as a by-product. This is followed by the transfer of the acyl intermediate to a nucleophilic substrate, including the *ε*-amino group of a peptide-bound lysine residue. Consequently, an intermolecular isopeptide *ε*-(*γ*-glutamyl)lysine bond is formed, which results in the internal cross-linking of monomeric protein units [27]. In transamidation reactions, lysine can be replaced by lower molecular mass amines, such as polyamines. Hence, in the presence of high concentration of polyamines, such as spermine, TG2 can form covalent cross-linking between two polypeptide chains, in the form of a dimer or an adduct as in the case of Gln–Gln or Gln–Gly- [27]. These bonds are resistant to chemical and physical degradation; hence, they are believed to be of biological significance especially in the stabilization of the extracellular matrix (ECM) [25, 28]. In cellular physiology, the isopeptide bonds formed by TG2 activity have been suggested to be functionally important in apoptosis, where they prevent inflammation by ensuring that the intracellular contents of dying cells are not released to the extracellular environment [29]. However, water can also act as a nucleophile and cause deamidation of protein-bound glutamine residues [27, 30]. The conversion of the acyl-donor glutamine residue to a glutamate residue triggers deamination. Originally, it was believed that the deamination reaction could only take place under conditions that do not favour transamidation [26, 31]. However, studies have reported the tendency of TG2 to carry out specific deamination [32], when structural features that favour deamination over transamidation are present in protein substrates [33].

## 3. Calcium-Independent Nonenzymatic Activities of Transglutaminase 2

In addition to the general hallmark of calcium-dependent transamidation activity, TG2 has other enzymatic activities, independent of Ca^2+^ as reviewed in Gundemir et al. [26]. For instance, TG2 can function as a protein kinase [24, 34–37], bind and hydrolyze GTP (GTPase and G-protein function) [11, 19, 22, 38–42], and exhibit protein disulfide isomerase activity* in vitro* [23] and* in vivo* [43–45], independent of calcium as reviewed in Belkin [25].

Over the past two decades, other functions of TG2 that are independent of its enzymatic activities have been established [46–52]. These calcium-independent, nonenzymatic and transamidation-independent activities of TG2 are involved in many critical physiological processes underlying many key aspects of cell behaviour, including cell adhesion, growth, migration, differentiation, programmed cell death, and ECM assembly [49]. In turn, these cellular processes are vital to embryogenesis and tissue morphogenesis, wound healing, and tissue repair, as well as tumor growth and metastasis [53].

In 1992, Gentile and colleagues first suggested the involvement of transglutaminase 2 in the mediation of extracellular matrix (ECM) adhesion [54]. They observed an astonishing effect of TG2 overexpression on the growth of fibroblasts and their increased resistance to trypsinization. Subsequent convincing proofs, at both the cellular and molecular levels, have supported TG2's involvement in the mediation of cellular interactions with the ECM and have demonstrated that TG2 serves as an adhesion receptor for fibronectin (FN) on the cell surface [46, 55–57].

Transglutaminase 2 has a very high affinity for FN, to which it has been shown to bind at a stoichiometry of 2 : 1 [58], independently of either Ca^2+^ or the transamidating and GTPase activities of TG2 [59]. The interaction of TG2 with FN has been shown to mediate ECM adhesion [46] and many other adhesion-dependent phenomena, such as cell migration, matrix assembly, and signalling [60, 61]. The gelatin-binding domain (42 kD) serves as the only binding site for TG2 on FN and binds TG2 with similar affinity as the whole FN molecule [62]. Additionally, the adhesive function of TG2 is favored by the fact that the 42 kD gelatin-binding domain of FN has no interaction sites for the numerous FN-binding integrins, as well as other FN-associated adhesion receptors [63]. Therefore, TG2 and integrin can independently bind distinct domains of FN, hence supporting a model of cooperation rather than engaging in competition in the cell adhesion process [49]. It has been shown in different cell types that the binding of TG2 to the 42 kD fragment of FN results in stable cell adhesion, limited spreading, and formation of specialized adhesive structures at the cell-substrate interface [56, 60].

Regardless of the coexistence of TG2 and integrin at different FN-binding domains, where they are involved in the cell adhesion process, TG2 also associates with integrin to maintain cell-extracellular matrix (ECM) interactions. Integrins represent a large class of transmembrane adhesion receptors constituted by distinct noncovalently associated and are composed of *α* and *β* subunits [64]. In all cell types apart from red blood cells, 24 integrin heterodimers constituted variously of 8 *β* subunits and 18 *α* subunits are expressed, serving as receptors for a number of ECM ligands and facilitate adhesion between cells [64, 65]. The role of integrin in wound healing, blood clotting and thrombosis, viral and bacterial infection, inflammation, tumor growth, and angiogenesis, as well as other pathological and physiological states, exemplifies the fundamental functions of integrin in cell-matrix adhesion [49].

Transglutaminase 2 has been shown to be associated with many integrin receptors, by binding to the extracellular domains of the *β*1 and *β*3 integrin subunits in different cell types [46, 56, 60]. The stable, noncovalent TG2-integrin complexes are formed independently of the transamidating activity of TG2, and there is no evidence of integrin serving as an enzymatic substrate of TG2 or other transglutaminases [46]. Furthermore, while performing some biochemical experiments on cells that do not synthesize FN, Akimov et al. [46], demonstrated that the TG2-integrin interaction is not mediated by fibronectin but is independent. They further observed that integrin-TG2 complexes have 1 : 1 stoichiometry and found that cell-surface TG2 is bound to integrin receptors, with the possibility of up to 40% of *β*1 integrin being associated with TG2 in various cell types [46, 60]. The ability of TG2 to form ternary adhesive complexes with various isoforms of integrin and FN, where all the three proteins successfully interact with each other, highlights the importance of TG2 in cell adhesion and indicates an unconventional role of TG2 as a coreceptor in cell-matrix interactions [46]. The implications of these nonenzymatic, calcium- and transamidation-independent activities in some key biological events are highlighted below.

## 4. Implications of Transglutaminase 2 in Biological Events: A Synoptic Update

Transglutaminase 2 is a multifunctional protein with over 130 substrates at various locations inside and outside the cell [66] (Table 2). The broad range of specificity of TG2 for its targets may account for its pleiotropic functionality. However, to achieve a particular function necessitates that the selection of a specific subset of protein substrate related to that particular biological event must be tightly regulated by additional factors. The various physiological implications of TG2 typify the relationships between its diverse biochemical activities and cellular functions and make it difficult to determine the exact role it plays in cell physiology and pathology.

### 4.1. Transglutaminase 2 in Cell Survival and Death Processes

With regard to cell death and survival, the role of TG2 is extremely complex, and for almost three decades it has remained under investigation, following the first report of TG2's involvement in apoptosis [67]. Transglutaminase 2's involvement in apoptosis could be better described as a double-edged sword as it can be both proapoptotic or antiapoptotic. Cells undergoing apoptosis show an increased level of TG2 expression, which may prime the cell to undergo apoptosis. Its inhibition results in a decreased propensity of cells to die by apoptosis [68, 69].

#### 4.1.1. Proapoptotic Activity of TG2

The proapoptotic activity of TG2 is defined by its cross-linking configuration, which requires a millimolar concentration of calcium. Stressful conditions, such as ultraviolet radiation and chemotherapeutic agents, can generate reactive oxygen species (ROS)—with resultant induction of TG2. Further increase in such stressful conditions may further trigger the release of Ca^2+^ from the endoplasmic reticulum (ER), resulting in the activation of TG2 and extensive cross-linking of intracellular proteins, which, in turn, initiates the apoptotic process [68, 70]. A major physiological significance of TG2 involvement in apoptotic initiation is its mediation of the crosstalk between dying and phagocytic cells to ensure tissue and cellular integrity. In essence, the focal function of TG2 in apoptosis is to ensure that, once the apoptotic process is initiated, it is completed without inflammation or tissue injury resulting from the process [71]. TG2 can achieve maintenance of a cellular environment devoid of inflammation whilst directly promoting apoptosis in some cell types [72] or indirectly promoting the activation of TGF-*β* released by the macrophages, which can promote the death of various cells [73, 74], to ensure that all damaged cells are killed quickly without the occurrence of necrosis. Additionally, TG2 can promote chemoattractant formation and the release of phosphatidylserine, to, respectively, aid macrophage migration to the site of apoptosis and the recognition of apoptotic cells [71, 75].

#### 4.1.2. Antiapoptotic Activity of TG2

The antiapoptotic effect of TG2 is independent of its transamidation and enzymatic cross-linking activities and does not require calcium. Nuclear TG2 protect cells from death by interacting with retinoblastoma protein pRb, polymerizing the alpha-inhibitory subunit of the transcription factor NF-kappa*β*, with the resultant transcriptional regulation of several antiapoptotic key genes [76]. Similarly, TG2 can translocate to the plasma membrane where it serves as a coreceptor for integrin, promoting its interaction with fibronectin. TG2-mediated interaction between integrin and fibronectin could result in the activation of cell survival and antiapoptotic signalling pathways, and extracellular matrix stabilization [68]. Also, in the extracellular space, TG2 can stimulate its own production by activating latent transforming growth factor beta (TGF-*β*), which in turn upregulates TG2 [71]. It is tempting to conclude that the proapoptotic and antiapoptotic effects of TG2 are dependent on the activation pathways and localization of the protein, with nuclear and extracellular TG2 as antiapoptotic and cytosolic TG2 is proapoptotic, in agreement with the findings of Milakovic et al. [77].

### 4.2. Transglutaminase 2 in Human Diseases

Owing to the pleiotropic and ubiquitous tissue distribution of TG2, it is not surprising that its involvement in many pathological conditions has been variously demonstrated. Transglutaminase 2 has been implicated as having a role in various chronic diseases, especially in (a) inflammatory diseases, including wound healing, tissue repair and fibrosis, and autoimmune diseases; (b) chronic degenerative diseases such as arthritis, atherosclerosis, and neurodegenerative conditions like Alzheimer's and Parkinson's diseases; (c) malignant diseases; and (d) metabolic diseases such as diabetes mellitus [28, 78]. In most of these diseases, the role of TG2 is mostly related to the dysregulation of its functions, especially regarding its interaction with, and stabilization of the cellular matrix, rather than its involvement in apoptosis.

#### 4.2.1. Transglutaminase 2 in Autoimmune Diseases

In autoimmune diseases such as coeliac disease, the presence of autoantibodies against TG2 and other substrates is an indication that TG2 may cross-link potential autoantigens to itself and to other protein substrates, triggering an immunological response typical for autoimmune diseases [79, 80]. TG2's role in coeliac disease is related to the deamination of the side chains of glutamine, in the presence of abundant glutamine in gluten proteins. This deamination reaction results in an upregulation of the binding capacity of gluten to DQ2 and the response of T-cell clones [81, 82]. Additionally, it has been reported that gluten peptides incubated with TG2 create covalent complexes through thioester bonds to the active site cysteine of TG2 and via isopeptide bonds to particular lysine residues of TG2 [83]. Hence, gluten proteins and their peptide derivatives serve as substrates for various TG2-catalysed reactions [78]. Recently, deamidation of gluten-derived gliadin peptides by TG2 was shown to be responsible for gliadin-induced toxicity and immune response in the small-intestinal mucosa [84]. Consequently, Rauhavirta and colleagues suggested that the inhibition of TG2 can reduce gliadin-induced effects [84]. In a different study, Oh et al. [85] reported that the initiation of allergen response in pulmonary epithelial cells requires TG2.

#### 4.2.2. Transglutaminase 2 in Inflammatory Diseases

In inflammatory diseases, TG2 plays a pivotal role via its regulatory action on granule secretion and macrophage function or by regulating the function of major inflammatory mediators like phospholipase A2 [86]. The involvement of TG2 in inflammatory diseases and related processes, such as angiogenesis and wound healing, has been reported [87, 88]. It is an important effector in the pathogenesis of chronic inflammatory diseases, like rheumatoid arthritis and osteoarthritis, by converting the latent transforming growth factor binding protein-1 into its active form, TGF-*β* [89]. Recently, TG2 has been reported to be directly involved in chronic kidney disease (CKD), where it is involved in the pathogenesis of vascular calcification through the enhancement of matrix vesicle-ECM interaction [90]. Similarly, on analysis of TG2 : creatinine ratio in relation to albumin : creatinine ratio in CKD patients, da Silva et al. [91] suggested that TG2 may be a potential biomarker for CKD detection and progression assessment.

#### 4.2.3. Transglutaminase 2 in Neurological and Metabolic Diseases

*In vitro* and/or* in vivo*, many TG2 substrates have been found in neuronal cellular compartments, for example, amyloid beta-A4 peptide, alpha synuclein, the microtubule-associated tau protein, synapsin I, and myelin basic protein, as reviewed by Facchiano et al. [78]. TG2-mediated cross-linking is therefore believed to be implicated in neurodegenerative diseases such as Huntington's, Alzheimer's, and Parkinson's diseases [79, 92] and in diseases related to neurotransmitter release [93]. Similarly, the possible involvement of TG2 in neurotransmitter release and related pathological conditions, such as that caused by tetanus neurotoxin intoxication, has been reported [94].

The covalent modification of TG2 substrates such as glyceraldehyde-3-phosphate dehydrogenase (GAPDH), alpha-ketoglutarate dehydrogenase, phosphoglycerate dehydrogenase, and fatty acid synthase [95], which are involved in energy metabolism, could account for the role of TG2 in metabolic diseases. Additionally, TG2-mediated covalent modification of hormone receptors or hormone-binding proteins indicates that TG2-catalysed cross-linking may be involved in controlling complex metabolic responses to hormones [96–98]. The involvement of TG2 in the regulation of insulin secretion and diabetes mellitus has also been suggested [99, 100].

#### 4.2.4. Transglutaminase 2 in Cancer

In cancer, transglutaminase 2 has been shown to play a major role in development of drug resistance and metastasis in many cancer types, including pancreatic carcinoma [101], ovarian carcinoma [102, 103], malignant melanoma [104], lung carcinoma [105], glioblastoma [106], and breast carcinoma [107]. When aberrantly regulated, TG2 could aid tumor cells to evade apoptosis and have direct consequences on cancer drug resistance [108, 109] and metastatic progression [107]. For instance, Park et al. [110] reported that TG2-specific cross-linking activity resulted in the polymerization and inhibition of nucleophosmin and concomitant increase in drug resistance potential of cancer cells. Recent evidence shows that aberrant expression of TG2 in mammary epithelial cells confers stem cell characteristics on the cells [111]. Similarly, Kumar and colleagues reported that high basal expression of TG2 in breast cancer cells promotes the development of stem cell features but did not mediate their terminal differentiation [111]. Additionally, Caffarel et al. [112] observed that the activation of TG2: integrin-*α*5*β*1 interactions through the stimulation of oncostatin M receptor in cervical squamous cell carcinoma induced promalignant changes.

Clinically, TG2 has been reported to serve as a predictive indicator of anticancer therapeutic efficacy. For instance, Jeong et al. [113] suggested that TG2 expression is a promising indicator of the effectiveness of epidermal growth factor receptor tyrosine kinase inhibitor (EGFR-TKI) therapy in patients suffering from nonsmall cell lung cancer. Similarly, Jasmeet et al. [114] reported that the accumulation of TG2 in tumour stroma can serve as an independent risk factor for the identification of invasive ductal carcinomas (IDCs) of breast and can establish breast cancer patients at high risk of recurrence. They also observed that overexpression of TG2 can serve as an indicator of poor prognosis for IDC of the breast. Agnihotri et al. [115] proposed that inflammation-induced progression of breast cancer and acquisition of survival and invasive capabilities by breast cancer cells are mediated by TG2. In acute myeloid leukaemia, Pierce et al. [116] demonstrated that increased expression of TG2 precipitated a more advanced state of the disease in relapse patients. They further established that increased TG2 expression correlates with the expression of proteins involved in apoptosis, motility, and extracellular matrix association and processes that have been linked with leukemia development and progression. This is a testament to the specialized ability of TG2 to interact with several proteins as substrates in various biological events, probably due to the unique biochemical structure of TG2 that is uncharacteristic of other transglutaminase enzymes.

## 5. Compensation for Transglutaminase 2 Functions

Regardless of the wide range of biological functionalities associated with TG2 and amidst its unique cellular biochemistry, its exact physiological functions are still debated. This debate is complicated by the fact that homozygous deletion of TG2 in mice does not result in an embryonic lethal phenotype [117, 118], suggesting that compensation for its absence may be achieved by other family members. Such knockouts are not however without associated pathology. For example, Bernassola et al. [100] observed that TG2-deficient mice displayed significant changes such as characteristic glucose intolerance and hyperglycaemia due to reduced insulin secretion, a condition equivalent to a subtype of diabetes called maturity-onset diabetes of the young (MODY). Moreover, a TG2-deficiency disease is yet to be identified in humans implying the importance of its presence.

This notion is further supported by the observation that TG2 is relatively more abundant than other members of the transglutaminase family. Its wide tissue distribution and its possession of a wide range of structural features that allows for flexibility in interaction with widely assorted proteins are some of the factors that give TG2 a potentially wide range of functions other than simple enzymatic activity.

Thus while there are increasing suggestions of possible compensation for the absence of TG2 by other members of the transglutaminase family [100], the pleiotropic nature of this protein indicates that TG2 is actually involved in more physiological processes than any other member of the enzyme family and, thus, while TG2 might feasibly compensate for other members of the family the suggestion that alternative TG isoforms may act as a universal “back up” system for TG2 seems less likely.

From the above, it seems more appropriate to argue that compensation for TG2's functions by other TGase isoforms might not be possible, except for roles that are determined by its calcium-dependent cross-linking and transamidating activities, which are common features of the transglutaminase family. For example, TG2-mediated functions that are enzymatic but independent of calcium, such as its role as a G-protein, protein disulphide isomerase activity, kinase function, and regulation of energy metabolism, are unlikely roles to be undertaken by any other member of the transglutaminase family. This could be due to absence of appropriate structural conformation in other transglutaminase enzymes that could enable them to alternately assume such roles in the event of TG2 absence.

Similarly, TG2-mediated integrin-fibronectin interaction is critical to many physiological events in the cell, including cell adhesion, growth, migration, differentiation, programmed cell death, and ECM assembly [25, 49]. Such interaction is vital to many cellular processes and serves as one of the major routes for extracellular survival, signalling activation, and consequent apoptotic evasion. It is nonenzymatic and independent of TG2 transamidation and cross-linking activities. Consequently, it is unlikely that any other member of the transglutaminase family can successfully compensate for this function in the event of TG2 absence.

## 6. Conclusions

The abundance of TG2 in various cell types, its specialized structural conformation, and its broad substrate specificity are some of the key factors justifying the enzyme's implication in myriads of biological events. From this review, it is evident that besides its calcium-dependent activities, TG2 can enzymatically or nonenzymatically mediate key cell physiological events. However, it has been increasingly suggested that in the event of TG2 absence its biological functions could be successfully compensated for by other members of the transglutaminase family. These suggestions have been made without recourse to the distinguishing features of TG2 among the transglutaminase family. It is necessary to carry out further investigations to ascertain the main reasons why TG2 knockout is not embryonic lethal, instead of relying on the assertion that its functions are compensated for by other transglutaminase enzymes. Finally, it is also our view that a systematic investigation should be carried out to establish, with certainty, the possibility of and premise for the replacement of TG2 function by any other member of the transglutaminase family.

## Figures and Tables

**Table 1:** Members of the transglutaminase (TGase) enzyme family, their nomenclature, tissue distribution, biological functions, and pathological involvement [4, 30].

TGase	Nomenclature	Tissue distribution, cellular and subcellular localization	Biological functions	Pathology
TG1	Keratinocyte TG, particulate TG, TG1, and TGK	Squamous epithelia, keratinocytes, cytosolic, membrane	Barrier function in stratified squamous epithelia, cornified envelope formation in keratinocyte differentiation	Lamellar ichthyosis [119]

TG2	Liver TG, tissue TG, cytosolic TG, erythrocyte TG, Gh*α*, and endothelial TG	Ubiquitously distributed in many types of tissue, cell membrane, cytosol, nucleus, extracellular	Apoptosis, cell survival signalling, cell differentiation, matrix stabilization, endocytosis, and so forth	Autoimmune diseases, neurodegenerative diseases, malignancies, metabolic diseases, and so forth [78]

TG3	Epidermal TG, callus TG, hair follicle TG, and bovine snout TG	Epidermis, hair follicle, brain, cytosolic	Terminal differentiation of keratinocytes, hair follicles	Human epidermis diseases

TG4	Prostate TG, TGp, androgen regulated major secretory protein, vesiculase, dorsal prostate protein 1 (DP1), and type 4 TG	Prostate gland, prostatic fluids, and seminal plasma, extracellular	Reproduction and fertility, especially in rodents where it is involved in semen coagulation	Not known

TG5	TGX, type 5 TG, and TG5	Ubiquitously expressed but predominant in female reproductive tissues, skeletal muscle, and foetal tissues, foreskin keratinocytes, epithelial barrier lining, cytosolic	Epidermal differentiation	Secondarily involved in hyperkeratotic phenotype in ichthyosis and in psoriasis, overexpressed or absent in different areas of the Darier's disease lesions [120]

TG6	TGY, type 6 TG, and TG6,	Testis, lungs, and brain, cellular localization is yet to be defined	Central nervous system development, motor function, and late stage cell envelope formation in the epidermis and the hair follicle	Spinocerebellar ataxias [121, 122]; polyglutamine (polyQ) diseases [123]

TG7	TGZ, type 7 TG, TG7, and transglutaminase 7	Ubiquitously expressed, prominent in testis and lungs, cellular and subcellular localization are unknown		Not known

FXIIIA	Factor XIII A, fibrin stabilizing factor, fibrinoligase, plasma TG, and Laki-Lorand factor	Chondrocytes platelets, placenta, astrocytes, macrophages, synovial fluid, heart, eye, bone, dendritic cells in the dermis	Wound healing, blood clotting, and bone growth	F13A1 deficiency characterized by impaired wound healing, spontaneous abortion in women, subcutaneous and intramuscular haematomas, and severe bleeding tendency

Band 4.2	Erythrocyte membrane protein band 4.2, B4.2, ATP-binding erythrocyte membrane protein band 4.2	Surface of erythrocyte membranes, bone marrow, foetal liver, spleen, membranal	Key component of erythrocyte skeletal network maintains erythrocyte shape and mechanical properties	Spherocytic elliptocytosis

**Table 2:** Transglutaminase 2 substrates, reactive sites, cellular localizations, and possible involvement in human physiology/diseases.

TG2 substrate	Reactive site	Localization	Physiology/disease
Acetylcholine esterase	Glutamine	Intracellular	Neurological disease [53]
Actin	Glutamine and lysine	Intracellular	Cytoskeleton regulation [124]
Aldolase	Reactive glutamine present but specific residue is unknown	Intracellular	Genetic disease, endocrine and metabolic diseases, autoimmune and inflammatory diseases [125]
Androgen receptor		Intracellular (nuclear receptor)	Endocrine and metabolic diseases [97, 98]
Annexin I (lipocortin I)	Glutamine	Intracellular	Autoimmune and inflammatory diseases, cytoskeleton regulation [126]
Calgizzarin-S100C protein-MLN 70—S100A11	Glutamine and lysine	Keratinocyte cornified envelope	Endocrine and metabolic diseases, dermatological diseases [127]
Collagen alpha 1(III)	Glutamine	Extracellular	Extracellular matrix interaction and stabilization, autoimmune and inflammatory diseases [128]
*α*-B-crystallin	Lysine	Intracellular	Cell life and death, cytoskeleton regulation, protein stabilization [129]
*β*-A3 crystallin	Glutamine	Intracellular	Cell life and death, cytoskeleton regulation, protein stabilization [130]
*β*-B3 crystallin	Glutamine	Intracellular	Cell life and death, cytoskeleton regulation, protein stabilization [131]
*β*-Bp (beta-B2) crystalline	Glutamine	Intracellular	Cell life and death, cytoskeleton regulation, protein stabilization [131]
Cytochrome c	Glutamine	Intracellular	Cell life and death [132]
Fibronectin	Glutamine	Extracellular	Protein stabilization, extracellular matrix interaction and stabilization [68]
Fibrinogen A alpha	Glutamine and lysine	Extracellular	Extracellular matrix interaction and stabilization, autoimmune and inflammatory diseases [133]
Glutathione S-transferase	Glutamine, lysine, and fluorescein	Intracellular	Extracellular matrix interaction and stabilization [134]
Gluten proteins	Glutamine	Extracellular	Celiac disease [79]
Glyceraldehyde 3 phosphate dehydrogenase	Lysine	Intracellular	Neurological diseases [135]
H3 histone	Glutamine	Intracellular	Cell life and death [136]
H4 histone	Glutamine	Intracellular	Cell life and death [136]
H2A histone	Glutamine	Intracellular	Cell life and death [136]
H2B histone	Glutamine	Intracellular	Cell life and death [136]
Importin alpha 3		Nuclear transport protein	Cell life and death [34]
*α*-Ketoglutarate dehydrogenase	Lysine	Intracellular	endocrine and metabolic diseases [137]
Latent TGF-beta binding protein-1 (LTBP-1)		Extracellular	Carcinogenesis, autoimmune, and inflammatory diseases [138]
*α*-2-Macroglobulin receptor-associated protein	Glutamine	Extracellular	autoimmune and inflammatory diseases [139]
Microtubule-associated protein tau-isoform Tau-F (Tau-4)	Glutamine and lysine	Intracellular	Cytoskeleton regulation, neurological diseases [140]
Myosin		Intracellular	Cytoskeleton regulation [141]
Nidogen	Glutamine	Extracellular	Extracellular matrix interaction and stabilization [142]
Osteocalcin		Extracellular	Autoimmune and inflammatory diseases [143]
Osteonectin	Glutamine	Extracellular	Autoimmune and inflammatory diseases, extracellular matrix interaction, and stabilization [144]
Osteopontin	Glutamine	Extracellular	Autoimmune and inflammatory diseases, extracellular matrix interaction, and stabilization [145]
Phospholipase A2	Glutamine	Extracellular	Endocrine and metabolic diseases, signal transduction, autoimmune, and inflammatory diseases [2, 86]
Troponin T		Intracellular	Cytoskeleton regulation [146]
